# Effects of Regular Low-Level Alcohol Consumption in Healthy Individuals: A Randomized, Double-Blind, Placebo-Controlled Crossover Study

**DOI:** 10.3390/healthcare10050882

**Published:** 2022-05-10

**Authors:** Shunji Oshima, Sachie Shiiya, Yasuhito Kato

**Affiliations:** Sustainable Technology Laboratories, Asahi Quality & Innovations, Ltd., 1-21 Midori 1-Chome, Moriya-shi 302-0106, Japan; sachie.siiya@asahi-qi.co.jp (S.S.); yasuhito.katou@asahi-qi.co.jp (Y.K.)

**Keywords:** low-level alcohol, biochemical indexes, general well-being schedule, liver function

## Abstract

The effects of alcohol consumption on health are suggested to depend on the amount of alcohol consumed. We investigated the objective and subjective health effects of the daily consumption of a small amount of alcohol in healthy individuals using a randomized, double-blind, placebo-controlled crossover study. Accordingly, 15 male and 27 female Japanese adults with average or lower general well-being schedule (GWBS) scores were asked to consume a beverage with 0.5% (*v*/*v*) alcohol (~4 g of alcohol a day; test beverage) and a placebo beverage two times daily for 4 weeks each. Regular low-level alcohol consumption significantly decreased the serum liver function indexes (aspartic aminotransferase, alanine aminotransferase, and lactate dehydrogenase) before and after consumption (*p* = 0.034, 0.033, and 0.013, respectively). The small amount of alcohol did not affect the participants’ GWBS scores; however, a stratified analysis with poor subjective well-being revealed that these changes differed significantly between low-level alcohol consumption and placebo-treated subjects (16.0 vs. 11.5, *p* = 0.041). In addition, changes in serum testosterone levels demonstrated a higher trend in the group that received the test beverage compared with the group that received the placebo beverage (*p* = 0.051). Daily low-level alcohol consumption may have positive effects on liver function and subjective well-being.

## 1. Introduction

The health effects of alcohol consumption are known to depend on the amount of alcohol consumed. Heavy drinking often results in health and social problems [1]. Many studies have reported on the J-shaped relationship between alcohol consumption and mortality [2,3,4,5,6], which indicates that light to moderate alcohol consumption is associated with health benefits relative to complete abstention or heavy alcohol use. In addition, the relationship between alcohol consumption and mental health has been documented [7]. Hazardous alcohol consumption can lead to poor mental health, but moderate alcohol intake is associated with a lower risk of incident depression [8]. Griswold et al. [9] reported that the risk of all-cause mortality, as well as that of cancer specifically, increased with increasing levels of consumption, and that the level of consumption that minimized health loss was zero (95% confidence interval = 0.0–0.8 standard drinks daily), without a J-shaped relationship observed. Further research is needed to elucidate the health effects of drinking different amounts of alcohol.

Nowadays, non-alcoholic beverages, which taste like alcoholic drinks, are a new trend and are widely available on the market because inadequate consumption of alcoholic drinks has been reported to produce health problems in consumers. Non-alcoholic is a regulatory term, and the laws regarding it vary across the globe. For example, in Japan, non-alcoholic beverages are treated as soft drinks, as their <1% alcohol content does not qualify them as alcoholic beverages under the Liquor Tax Law. Although some drinks that fall under the category of non-alcoholic beverages undoubtedly contain alcohol, at least as far as we know, the health effects resulting from their consumption have not been reported to date. Even a very small amount of alcohol has been proposed to have some physiological effects. Research has also suggested the lowest mortality rate for intake of <5 g of alcohol per day on the J-shaped curve [10,11]. Several animal studies that investigated the effects of low-level alcohol consumption in rats or mice that were given drinking water with 1% alcohol did not find harmful effects [12,13,14,15,16]. Moreover, these studies reported an amelioration of certain parameters, supporting the notion that low-level alcohol drinking might exhibit beneficial health effects. In one animal study, the plasma alcohol levels of Wistar rats that received 1% alcohol oscillated between 0.3 and 0.5 mmol/L [12].

We hypothesized that lower blood alcohol levels are the concentrations reached by the consumption of a non-alcoholic beverage with a small amount of alcohol that can be easily exceeded as a result of light to moderate alcohol consumption in humans. We surmised that 0.5% (*v*/*v*) alcohol (0.5%Alc) is suitable to reach 0.3 to 0.5 mmol/L of blood alcohol levels based on a previous report [17]. This study aimed to exploratively evaluate the effects of daily low-level alcohol consumption on objective (various biochemical indexes) and subjective (well-being) health. As described by the Constitution of the World Health Organization, health is a state of complete physical, mental, and social well-being and not merely the absence of disease or infirmity. Well-being indicates being in a good physical, mental, and social condition. Diener and Chan reported evidence suggesting that subjective well-being causally affects health and longevity [18]. To this end, we conducted a randomized, double-blind, placebo-controlled crossover clinical study with healthy individuals who were assigned to one of the following two groups: group A, who were given a beverage with 0.5%Alc (<4 g of alcohol a day) first and then a placebo beverage for 4 weeks each, or group B, who were given a placebo beverage first and then a beverage with 0.5%Alc for 4 weeks each. This was, to the best of our knowledge, the first trial to have examined the health effects of low-level alcohol consumption.

## 2. Materials and Methods

### 2.1. Participants

Healthy Japanese male and female adults aged between 40 and 69 years and who demonstrated a general well-being schedule (GWBS) score of <70 were recruited for the study. The GWBS is a widely used and self-administered questionnaire that measures subjective well-being [19], with lower GWBS scores reflecting lesser subjective well-being. The average GWBS score in the Japanese population is almost 70 points [20]. Therefore, this criterion suggested the trial excluded volunteers with good subjective well-being. Individuals with a drinking habit (more than one drinking session per week), who were undergoing drug treatment as a patient, who presented with a drug or alcohol dependence, who presented with a drug or food allergy or who routinely used drug or dietary supplements for alcohol metabolism, and those who were pregnant or lactating were excluded from the study. All participants provided written informed consent before participating. Of the 106 participants who underwent screening tests, 42 were enrolled according to the above-cited inclusion and exclusion criteria.

### 2.2. Study Beverages

Water containing 0.5%Alc (test beverage) and water (placebo beverage) (465 mL) was manufactured by a beverage manufacturing consignment company for the clinical trial. All participants ingested both steel-canned test beverages, which had the same appearance. Small amounts of lemon flavor and citric acid were added to the two study beverages so that they could not be distinguished from each other. We confirmed that, when ingesting the beverage containing 0.5%Alc, the alcohol could not be sensually tasted and it did not cause even the slightest feeling of inebriation.

### 2.3. Study Design

**Figure 1:**
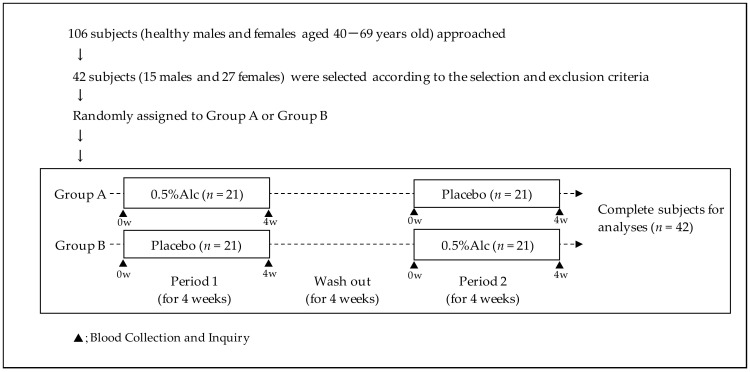
Schedule of the randomized, double-blind, placebo-controlled crossover clinical study. The study was performed over a 12-week period.

This randomized, double-blind, placebo-controlled crossover study was registered with the University Hospital Medical Information Network (UMIN-CTR; registration ID: UMIN000044998). The study schedule is shown in Figure 1. Forty-two participants were randomly allocated to one of the two study groups. In detail, a computer-generated stratified randomized schema was used to assign the participants to either group A or group B, with matching based on age, sex, and GWBS score. Group A consumed the test beverage during study period 1 and the placebo beverage during study period 2, whereas group B received the regimen in reverse sequence (i.e., placebo then 0.5%Alc). All participants consumed the study beverages two times (morning and evening) daily and were instructed to maintain their daily eating habits and normal levels of daily physical activity during the study period of 12 weeks (study period 1, washout, and study period 2). Peripheral blood specimens from the participants were collected from the cubital vein at the start and end of the day in periods 1 and 2, respectively, so as to determine each blood parameter. Furthermore, all participants answered the GWBS and Pittsburgh Sleep Quality Index (PSQI) as subjective questionnaires.

### 2.4. Blood Analyses and Subjective Assessments

Serum aspartic aminotransferase (AST), alanine aminotransferase (ALT), lactate dehydrogenase (LDH), γ-glutamyl transferase (GGT), alkaline phosphatase (ALP), total bilirubin, creatinine, blood urea nitrogen (BUN), low-density lipoprotein cholesterol (LDL-C), high-density lipoprotein cholesterol (HDL-C), triglyceride, uric acid, total protein, albumin, free testosterone, dehydroepiandrosterone sulfate (DHEA-S), plasma cortisol, adrenocorticotropic hormone (ACTH), and blood glucose were measured by a local clinical laboratory (SRL, Tokyo, Japan).

GWBS and PSQI were used in the study to evaluate the subjective quality of life. The 17-item version of the GWBS was used [20]. Briefly, the 17 items included the following: (1) general feeling; (2) nervousness; (3) firm control; (4) sad, discouraged, or hopeless; (5) strain, stress, or pressure; (6) happy and satisfied with life; (7) afraid of losing one’s mind or control; (8) anxious, worried, or upset; (9) waking up fresh and rested; (10) bothered by bodily disorders; (11) down-hearted and blue; (12) emotionally stable and confident; (13) feeling tired and worn out; (14) concerned/worried; (15) relaxed–tense; and (16) energy level, pep, or vitality; and (17) cheerful–depressed. This study used the Japanese version of the questionnaires without changes, as described in the literature [21]. All participants were required to select one response from six possible answers for each item that best described their feelings over the previous four weeks. This 17-item version indicated adequate reliability and concurrent validity similar to the General Health Questionnaire, aimed at detecting current psychiatric disturbance [22]. Its adequacy as a comprehensive assessment tool for well-being has also been reported for the Japanese population [20].

PSQI assesses sleep quality and disturbances over the course of 1 month [23]. From the 19 individual items, seven component scores were generated, namely, subjective sleep quality (one item), sleep latency (two items), sleep duration (one item), habitual sleep efficiency (three items), sleep disturbances (nine items), use of sleeping medication (one item), and daytime dysfunction (two items). All participants were required to select a number (0–3) that best described their feelings over the previous four weeks. The sum of scores for these seven components, which yields one global score, was assessed in this study and ranged from 0 to 21 points, with lower scores indicating better sleep quality. The Japanese version of the questionnaires was used without alterations, as described in the literature [24].

### 2.5. Statistical Analysis

All statistical analyses were performed using BellCurve for Excel 3.20 (Social Survey Research Information, Tokyo, Japan). The Shapiro–Wilk test was used to determine the normality of data. Accordingly, parametric paired *t*-tests were conducted when the normality assumptions were satisfied; otherwise, the non-parametric sign test was used. Temporal changes for each parameter from before consumption (baseline) to 4 weeks later and the differences in temporal changes for each parameter between the study groups were analyzed using paired *t*-tests; data were expressed as mean (standard deviation) for parametric analyses. Differences in changes in baseline GWBS and PSQI scores between the study groups were analyzed using sign tests; data were expressed as median (interquartile range) for the non-parametric analyses. Differences were considered significant when the probability of no difference was <5% and non-significant when the probability of no difference was <10%.

## 3. Results

This study had no dropouts at any point during the 12-week crossover period. Thus, final analyses were performed using data from 42 participants (15 men and 27 women). However, as a female participant from group A demonstrated difficulty undergoing blood collection, statistical analyses for blood parameters were conducted using data from all of the other participants only. None of the participants drank alcoholic beverages during the study. Two male participants were current smokers. Significant changes in body weight and body mass index before and after the consumption of 0.5%Alc or placebo for 4 weeks were not observed. The characteristics of the study participants are detailed in Table 1.

The changes in each of the blood parameter concentrations during the trial are summarized in Table 2. The AST, ALT, and LDH levels after 4 weeks were significantly lower than those before consumption in the 0.5%Alc group, whereas changes in these parameters were not observed in the placebo group. The changes in AST (*p* = 0.059) and ALT (*p* = 0.064) levels in the 0.5%Alc group indicated a non-significant low trend relative to those in the placebo group.

**Figure 2:**
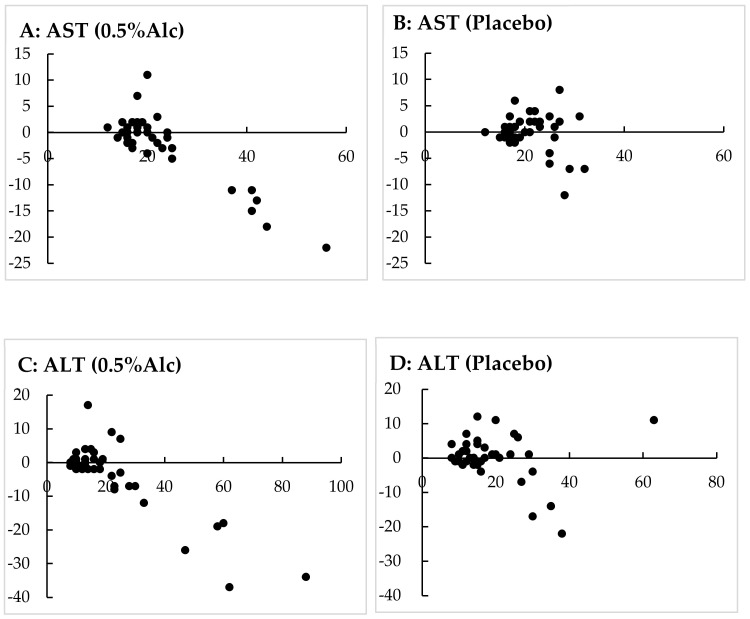
Relationships between the baseline levels and varying amounts of serum AST and ALT. The X-axis shows the baseline serum AST or ALT concentration (units per liter), whereas the Y-axis shows the varying amounts of serum AST or ALT concentration (units per liter) from baseline to 4 weeks later. (**A**) Serum AST levels of the 0.5%Alc group. (**B**) Serum AST levels of the placebo group. (**C**) Serum ALT levels of the 0.5%Alc group. (**D**) Serum ALT levels of the placebo group (*n* = 41). AST, aspartic aminotransferase; ALT, alanine aminotransferase.

Figure 2 illustrates the relationships between the baseline levels of serum AST and ALT and varying amounts of those levels. The participants from the 0.5%Alc group who presented with a high serum AST or ALT level before consumption exhibited a lower concentration after consumption, whereas this trend was not observed in the placebo group. Serum total protein levels after 4 weeks were significantly lower than those before consumption in the placebo group, but changes in these parameters were not observed in the 0.5%Alc group. Moreover, changes in serum GGT, ALP, total bilirubin, creatinine, BUN, LDL-C, triglyceride, albumin, and blood glucose levels were not observed before and after consumption in either study group. HDL-C and uric acid levels after 4 weeks exhibited a non-significant high trend compared with those before consumption in the 0.5%Alc group.

The changes in GWBS and PSQI scores as a result of the consumption of the study beverages are summarized in Table 3. Varying scores from baseline did not significantly differ between the study groups (*p* = 0.206). Additionally, stratified analyses were performed according to the previously reported criteria [21]. Analyses of 30 participants with a GWBS score of ≤60, which indicated a poor subjective well-being, revealed that varying GWBS scores in the 0.5%Alc group were significantly higher than those in the placebo group (*p* = 0.041). Differences in varying PSQI scores from baseline were not observed between the groups (*p* = 1.000).

Serum-free testosterone, DHEA-S, plasma cortisol, and ACTH concentrations as endocrinological parameters were measured as shown in Table 4. Changes in these concentrations from baseline did not significantly differ between the study groups. A further stratified analysis of the data from the 30 participants with a poor subjective well-being found that the changes in free testosterone concentrations from baseline to 4 weeks later in the 0.5%Alc group demonstrated a non-significant increasing trend relative to the placebo group (*p* = 0.051), although the DHEA-S, cortisol, and ACTH levels did not differ between the study groups.

## 4. Discussion

Little is known about the health effects of low-level alcohol (<1%) consumption. This study aimed to exploratorily evaluate the effects of daily low-level alcohol consumption on objective and subjective health. We conducted our clinical trial using two study beverages, namely, a beverage with 0.5%Alc (~4 g of alcohol a day) and a placebo beverage that healthy participants consumed for 4 weeks each. This was, to the best of our knowledge, the first trial that examined the health effects of low-level alcohol consumption.

Surprisingly, the serum AST, ALT, and LDH levels after regular consumption were significantly lower than those before consumption in the 0.5%Alc group. In particular, the participants from the 0.5%Alc group who presented with a high serum AST or ALT level before consumption demonstrated a lower concentration of the corresponding parameter being measured after consumption (see Figure 2). Aminotransferases such as AST and ALT are excellent markers related to hepatocellular injury, and LDH is a well-known parameter of damage to several tissues, including those in muscles and the heart, apart from liver damage. Alcoholic liver disease has been well documented to result from the dose- and time-dependent consumption of alcohol [25].

Alcohol-induced oxidative stress has been suggested to play a major role in the mechanisms through which alcohol causes liver damage [26,27,28]. The induction of cytochrome P4502E1 (CYP2E1) by alcohol has been proposed as a central pathway that contributes to the ability of alcohol to induce oxidative stress. CYP2E1 metabolizes ethanol, and CYP2E1 levels are elevated after moderate and heavy alcohol consumption. CYP2E1 is also an effective generator of some reactive oxygen species. However, heavy low-level alcohol consumption in rats has been reported to augment a reduced glutathione/oxidized glutathione ratio and lipid peroxide levels in the liver, with the liver CYP2E1 activity being unchanged [12]. A small dose of alcohol can potentially mitigate oxidative stress in humans. Unfortunately, we did not try to measure oxidative stress markers in this study. Further studies are required in order to elucidate the ameliorative effects and mechanisms of low-level alcohol consumption in liver function. Alcohol consumption is widely known to lead to increased serum uric acid [29] and HDL-C [30] levels. However, as far as the available data are concerned, the impact of low-level alcohol consumption on these parameters is considered to be minor.

In relation to subjective health scores based on GWBS (for well-being) and PSQI (for sleep conditions), this study did not find differences between the 0.5%Alc and placebo groups overall. However, the stratified subgroup analysis, which predicted poor well-being, revealed that the changes in GWBS scores from baseline in the 0.5%Alc group were superior to those in the placebo group. GWBS measures subjective well-being and distress; it rates items concerning an individual’s psychological state during the previous month, with the total score representing one’s comprehensive subjective well-being [19]. Higher GWBS scores indicate greater subjective well-being. Substantial evidence has indicated that high subjective well-being promotes better health and longevity [18]. In fact, an increase in the GWBS score is associated with reduced odds of an abnormal T-axis, which is associated with fatal and nonfatal cardiac events and a lower hazard of composite cardiovascular disease hospitalization and death [31]. Thus, an increase in GWBS score may be expected to provide health benefits. Many factors are considered to affect subjective well-being [32]. We examined testosterone [33,34], DHEA-S [35,36], cortisol [37], and ACTH [38] as endocrinological factors that could potentially affect changes in subjective well-being. Our total and stratified analyses determined that DHEA-S, cortisol, and ACTH did not differ between the study groups. However, for the 30 participants with a poor subjective well-being, varying levels of serum-free testosterone in the 0.5%Alc group demonstrated a higher trend compared with those in the placebo group. Nevertheless, the analysis of all of the participants did not indicate a difference between the study groups. Previous reports have suggested that alcohol consumption affects the testosterone levels of both men and women. The plasma testosterone concentration is likely to decrease during hangovers and alcohol withdrawal [39]. A moderate dose (0.34–0.5 g/kg) of alcohol has been found to temporally increase plasma testosterone levels in healthy men [40] and healthy premenopausal women [41]. Alcohol metabolism produces a significant increase in the NADH/NAD+ (nicotinamide adenine dinucleotide hydrogen/nicotinamide adenine dinucleotide+) ratio in the cytoplasm and mitochondria, as evidenced by an increase in the lactate/pyruvate ratio [42]. The NAD+ dependent metabolism of alcohol due to alcohol dehydrogenases has also been reported to inhibit the NAD+-dependent 3-beta-hydroxysteroid dehydrogenase reaction, resulting in the inhibition of testosterone synthesis. Conversely, supplementation with pyruvate has been shown to prevent the alcohol-induced inhibition of testosterone synthesis in Leydig cells [43]. Our unpublished data for another clinical trial indicate that changes in blood pyruvate levels after the consumption of 0.5%Alc significantly increased relative to those after the consumption of water. Testosterone replacement has been reported to improve well-being in women and men [44]. Regular low-level alcohol consumption may reinforce testosterone synthesis because of the acceleration of pyruvate generation in the case of lower testosterone levels. Further studies are required to elucidate the effects and mechanisms of daily low-level alcohol consumption in subjective well-being.

Experimental and clinical studies have shown that alcohol use affects sleep physiology and subjective reports of insomnia symptoms [45]. PSQI has often been used as an outcome measure in clinical trials of interventions intended to reduce sleep disturbances, with a score of ≥6 indicating poor sleep quality [46]. In this study, 20 and 17 participants from the 0.5%Alc group and placebo group, respectively, exhibited poor sleep quality before consumption. After consumption, only two participants from the 0.5%Alc group and three from the placebo group experienced deteriorated sleep conditions. These outcomes confirm that a small amount of alcohol does not affect sleep status. As we expected, during this clinical trial, low-level alcohol consumption did not lead to adverse effects. Based on this result, the daily consumption of a beverage with 0.5%Alc does not seem to negatively affect health in the short term. In reality, many individuals consume a small amount of alcohol daily through non-alcoholic beverages that contain only a few grams of alcohol. To the best of our knowledge, the findings of this study are the first to suggest the health effects of low-level alcohol consumption.

With regard to limitations, this study set the consumption period of crossover regimens to only 4 weeks. Longer trials may show statistically greater benefits of low-level alcohol consumption, and such studies would be best performed as parallel-group comparisons. In addition, a power analysis for calculating the required number of study participants was not performed during the planning phase of the study—because of its exploratory research design. Evidence for each effect of low-level alcohol consumption could be acquired by conducting clinical trials with the appropriate number and characteristics of participants. Overall, further studies are required to clarify the health effects of low-level alcohol consumption.

## Figures and Tables

**Table 1:** Characteristics of the study participants (*n* = 42).

Parameter	Mean (SD)
Age (y)	52.3 (7.3)
Male, *n* = 15	53.1 (7.9)
Female, *n* = 27	51.8 (7.0)
Height (cm)	164.3 (8.9)
Male, *n* = 15	173.1 (6.5)
Female, *n* = 27	159.4 (5.7)
Body weight (kg)	55.3 (8.5)
Male, *n* = 15	62.2 (7.8)
Female, *n* = 27	51.5 (6.2)
Body mass index (kg/m^2^)	20.4 (2.2)
Male, *n* = 15	20.7 (2.1)
Female, *n* = 27	20.2 (2.2)
Systolic blood pressure (mmHg)	113 (16)
Diastolic blood pressure (mmHg)	75 (9)
Hemoglobin (g/dL)	13.7 (1.3)
Hematocrit (%)	42.4 (3.6)
Platelet count (10^4^/μL)	24.1 (5.5)
White blood cell count (10^4^/μL)	5269 (1306)
Red blood cell count (10^4^/μL)	448 (34)

Each measurement was obtained at the initial screening.

**Table 2:** Changes in blood parameter concentrations during the crossover trial.

Parameter	Study Group	Before Consumption	After 4 Weeks	*p* ^a^	Change from	*p* ^b^
					Baseline	
AST (IU)	Placebo, *n* = 41	21 (5)	21 (5)	0.965	−0 (4)	0.059 ^†^
	0.5%Alc, *n* = 41	22 (9)	20 (5)	0.034 *	−2 (6)	
ALT (U/L)	Placebo, *n* = 41	19 (10)	19 (11)	0.838	−0 (7)	0.064 ^†^
	0.5%Alc, *n* = 41	22 (17)	18 (10)	0.033 *	−4 (10)	
LDH (U/L)	Placebo, *n* = 41	173 (26)	172 (23)	0.576	−1 (14)	0.340
	0.5%Alc, *n* = 41	173 (27)	169 (25)	0.013 *	−4 (10)	
GGT (U/L)	Placebo, *n* = 41	21 (11)	20 (9)	0.400	−1 (6)	0.718
	0.5%Alc, *n* = 41	22 (14)	22 (12)	0.453	−1 (5)	
ALP (U/L)	Placebo, *n* = 41	72 (20)	71 (20)	0.154	−1 (6)	0.270
	0.5%Alc, *n* = 41	72 (21)	73 (22)	0.603	1 (10)	
Total bilirubin (mg/dL)	Placebo, *n* = 41	0.7 (0.3)	0.7 (0.2)	0.704	−0.0 (0.2)	0.184
	0.5%Alc, *n* = 41	0.7 (0.3)	0.6 (0.2)	0.130	−0.1 (0.2)	
Creatinine (mg/dL)	Placebo, *n* = 41	0.7 (0.2)	0.7 (0.2)	0.140	−0.0 (0.0)	0.322
	0.5%Alc, *n* = 41	0.7 (0.2)	0.7 (0.1)	0.944	−0.0 (0.0)	
BUN (mg/dL)	Placebo, *n* = 41	13.9 (2.9)	13.4 (3.5)	0.264	−0.5 (2.9)	0.150
	0.5%Alc, *n* = 41	13.7 (3.2)	14.2 (3.3)	0.301	0.6 (3.4)	
Glucose (mg/dL)	Placebo, *n* = 41	88 (7)	89 (7)	0.886	0 (8)	0.837
	0.5%Alc, *n* = 41	89 (6)	90 (9)	0.661	1 (8)	
LDL-C (mg/dL)	Placebo, *n* = 41	125 (23)	122 (20)	0.179	−3 (13)	0.113
	0.5%Alc, *n* = 41	120 (22)	123 (20)	0.237	2 (12)	
HDL-C (mg/dL)	Placebo, *n* = 41	71 (14)	72 (14)	0.703	0 (6)	0.276
	0.5%Alc, *n* = 41	69 (13)	71 (15)	0.077 ^†^	2 (7)	
Triglyceride (mg/dL)	Placebo, *n* = 41	73 (33)	72 (28)	0.722	−1 (21)	0.416
	0.5%Alc, *n* = 41	77 (37)	72 (41)	0.235	−5 (27)	
Uric acid (mg/dL)	Placebo, *n* = 41	4.7 (1.1)	4.7 (1.1)	0.740	0.0 (0.5)	0.303
	0.5%Alc, *n* = 41	4.6 (1.3)	4.8 (1.3)	0.062 ^†^	0.2 (0.5)	
Total protein (g/dL)	Placebo, *n* = 41	7.3 (0.4)	7.1 (0.4)	0.006 *	−0.1 (0.3)	0.283
	0.5%Alc, *n* = 41	7.2 (0.3)	7.1 (0.3)	0.116	−0.1 (0.2)	
Albumin (g/dL)	Placebo, *n* = 41	4.4 (0.3)	4.4 (0.3)	0.474	−0.0 (0.2)	0.700
	0.5%Alc, *n* = 41	4.4 (0.3)	4.3 (0.2)	0.183	−0.0 (0.2)	

Data are presented as mean (standard deviation). Differences were considered significant when * *p* < 0.05 and non-significant when ^†^ *p* < 0.1. AST, aspartic aminotransferase; ALT, alanine aminotransferase; LDH, lactate dehydrogenase; GGT, γ-glutamyl transferase; ALP, alkaline phosphatase; BUN, blood urea nitrogen; LDL-C, low-density lipoprotein cholesterol; HDL-C, high-density lipoprotein cholesterol. ^a^ Paired *t*-test comparing levels measured before consumption with those recorded 4 weeks later. ^b^ Paired *t*-test of changes from baseline in the study groups.

**Table 3:** Comparative results for GWBS and PSQI scores between the study groups.

Subjective	Study Group	Before Consumption	After 4 Weeks	Change from	*p* ^a^
Parameter				Baseline	
GWBS	Placebo, *n* = 42	54.5 (13.5)	67.0 (17.5)	12.0 (13.0)	0.206
	0.5%Alc, *n* = 42	55.0 (19.5)	70.0 (14.8)	15.0 (16.5)	
	Stratified analyses				
	Placebo, *n* = 30	53.0 (11.8)	62.5 (10.5)	11.5 (11.8)	0.041 *
	0.5%Alc, *n* = 30	49.5 (15.0)	67.0 (16.8)	16.0 (19.5)	
PSQI	Placebo, *n* = 42	5.0 (4.5)	4.0 (3.0)	−1.0 (2.0)	1.000
	0.5%Alc, *n* = 42	5.0 (5.0)	4.0 (3.0)	−1.0 (1.8)	

Data are presented as median (interquartile range). Differences were considered significant when * *p* < 0.05. Stratified analyses were performed according to the criterion of having a GWBS score of ≤60, which predicted poor subjective well-being. GWBS, general well-being schedule; PSQI, Pittsburgh Sleep Quality Index. ^a^ Sign test of changes from baseline in the study groups (total analyses, *n* = 42; stratified analyses, *n* = 30).

**Table 4:** Comparative results for endocrinological parameters between the study groups.

Parameter	Study Group	Before Consumption	After 4 Weeks	Change from	*p* ^a^
				Baseline	
Free testosterone (pg/mL)	Placebo, *n* = 41	4.8 (5.7)	4.9 (6.0)	0.14 (2.0)	0.973
	0.5%Alc, *n* = 41	4.6 (5.3)	4.9 (5.7)	0.15 (1.8)	
	Stratified analyses				
	Placebo, *n* = 30	4.8 (6.0)	4.5 (5.7)	−0.4 (1.2)	0.051 ^†^
	0.5%Alc, *n* = 30	4.3 (5.3)	4.7 (5.8)	0.2 (2.0)	
DHEA-S (μg/dL)	Placebo, *n* = 41	129 (60)	126 (63)	−3 (25)	0.903
	0.5%Alc, *n* = 41	127 (59)	123 (58)	−4 (31)	
	Stratified analyses				
	Placebo, *n* = 30	118 (62)	110 (59)	−8 (23)	0.579
	0.5%Alc, *n* = 30	111 (54)	107 (54)	−4 (30)	
Cortisol (μg/dL)	Placebo, *n* = 41	7.4 (1.8)	7.0 (2.4)	−0.3 (2.5)	0.131
	0.5%Alc, *n* = 41	7.0 (1.8)	7.5 (2.6)	0.5 (2.6)	
	Stratified analyses				
	Placebo, *n* = 30	7.4 (1.9)	7.1 (2.5)	−0.3 (2.5)	0.142
	0.5%Alc, *n* = 30	6.7 (1.5)	7.4 (2.7)	0.6 (2.4)	
ACTH (pg/mL)	Placebo, *n* = 41	19.9 (11.9)	19.6 (11.5)	−0.3 (8.2)	0.687
	0.5%Alc, *n* = 41	19.5 (11.0)	19.8 (12.1)	0.3 (5.3)	
	Stratified analyses				
	Placebo, *n* = 30	19.6 (12.1)	19.8 (12.7)	0.2 (8.6)	0.872
	0.5%Alc, *n* = 30	17.9 (10.8)	18.6 (11.8)	0.4 (4.2)	

Data are presented as mean (standard deviation). Differences were considered significant when *p* < 0.05 and non-significant when ^†^ *p* < 0.1. Stratified analyses were performed according to the criterion of having a GWBS score of ≤60, which predicted a poor subjective well-being. DHEA-S, dehydroepiandrosterone sulfate; ACTH, adrenocorticotropic hormone. ^a^ Paired *t*-test of changes from baseline in the study groups.

## Data Availability

The datasets used in this study are available from the corresponding author upon reasonable request.
